# Facilitation of Crossmodal Integration During Emotional Prediction in Methamphetamine Dependents

**DOI:** 10.3389/fncir.2019.00080

**Published:** 2020-01-23

**Authors:** Zhao Zhang, Weiqi He, Yuchen Li, Mingming Zhang, Wenbo Luo

**Affiliations:** Research Center of Brain and Cognitive Neuroscience, Liaoning Normal University, Dalian, China

**Keywords:** methamphetamine dependents, visual–auditory integration, emotion recognition, facial expression, voice

## Abstract

Methamphetamine (meth) can greatly damage the prefrontal cortex of the brain and trigger dysfunction of the cognitive control loop, which triggers not only drug dependence but also emotional disorders. The imbalance between the cognitive and emotional systems will lead to crossmodal emotional deficits. Until now, the negative impact of meth dependence on crossmodal emotional processing has not received attention. Therefore, the present study firstly examined the differences in crossmodal emotional processing between healthy controls and meth dependents (MADs) and then investigated the role of visual- or auditory-leading cues in the promotion of crossmodal emotional processing. Experiment 1 found that MADs made a visual–auditory integration disorder for fearful emotion, which may be related to the defects in information transmission between the auditory and auditory cortex. Experiment 2 found that MADs had a crossmodal disorder pertaining to fear under visual-leading cues, but the fearful sound improved the detection of facial emotions for MADs. Experiment 3 reconfirmed that, for MADs, A-leading cues could induce crossmodal integration immediately more easily than V-leading ones. These findings provided sufficient quantitative indicators and evidences that meth dependence was associated with crossmodal integration disorders, which in turn was associated with auditory-leading cues that enhanced the recognition ability of MADs for complex emotions (all results are available at: https://osf.io/x6rv5/). These results provided a better understanding for individuals using drugs in order to enhance the cognition for the complex crossmodal emotional integration.

## Introduction

Multisensory integration refers to the convergence or integration of multiple sensory signals into a biologically internal representation, which can maximize the effectiveness of information transmission in the ecological environment and improve the ACC of perception (Parise and Ernst, 2016). Emotional VA integration is an effective way to transmit and decode emotional information (Zhang H. et al., 2018), and it is of great significance to human survival and evolution. Previous researchers have revealed the neural mechanisms of emotional VA integration preliminarily. For instance, Bruck et al. (2011) proposed the neurobiological model of multisensory emotional information processing. Specifically with perceptual processing as the first stage, the emotional VA information is extracted within modality-specific primary and secondary cortices. In the second stage, the emotional VA information is transmitted to the posterior superior temporal lobe, where a single emotional perception forms. Subsequently, the cognition and evaluation of emotion are accomplished in the DLPFC and OFC (Johnson et al., 2007).

Mood disorders accompany drug use (Thoma et al., 2013; Attwood and Munafo, 2014). For example, depression is three to four times more prevalent among drug addicts than non-drug ones (Lai et al., 2015). Methamphetamine (meth) is one of the most widely used illegal drugs in the world. Meth dependents (MADs) are generally associated with emotional issues (Kim et al., 2011; Chen et al., 2018). Emotional processing mainly refers to recognizing the facial expressions of others, which forms the basis of social interaction (Becker et al., 2012). Researches have shown that drug use can destroy social relationships, which is closely related to the damaged recognition or decoding of facial expressions (Volkow et al., 2011a; Heilig et al., 2016; Cox et al., 2018; Nummenmaa and Tuominen, 2018). Functional neuroimaging studies have found that when exposed to emotional images, MADs show abnormal activation mainly in the prefrontal and other limbic-related regions (e.g., the amygdala) (Uhlmann et al., 2016), compared with HCs (Payer et al., 2008; Kim et al., 2011), which plays an important role in emotional regulation. It indicates that the emotional defects of MADs should be due to the dysfunctional cognition or motivation or the reduced activity in the cognitive control circuit (Volkow et al., 2011a; Zhang L. et al., 2018). Therefore, it is very necessary to further discuss the emotional disorders of MADs.

In addition, the PFC and olfactory play a key role in the convergence of crossmodal signals (Klasen et al., 2012; Yalachkov et al., 2012). Wang et al. (2018) reported the abnormal functional connection between the left OFC and DLPFC in an alcohol-dependent group. These results suggest that alcoholism and MADs may share a similar neural mechanism linked to crossmodal. The dual-process model suggests that the reflective system based on the frontal cortex is mainly responsible for memory, executive functions, and cognitive evaluation; the affective-automatic system based on the limbic regions is responsible for emotional evaluation (Noel et al., 2010; Wiers et al., 2013), indicating that addictive behavior may be associated with both cognitive deficits and an imbalance between the reflective and affective-automatic system. Moreover, crossmodal cues have high ecological validity. Therefore, it is of great significance to study the emotional crossmodal defects of MADs.

Recently, Chen et al. (2018) investigated the dysfunction of emotional VA integration in MADs. They used neutral and emotional videos for startle reflex testing. MADs exhibited a lower subjective arousal level of fear and enhanced startle response to anger, suggesting that the crossmodal disorder on negative emotion might illustrate its correlation with MADs. However, several limitations still remain. In the above study, neither the emotional crossmodal with common behavioral indices such as RT or ACC nor differences in behavioral indicators were quantified, namely, compared to unisensory, crossmodal processing could serve to increase ACC or shorten RTs (Ball et al., 2018). These cases may make an underestimate of the crossmodal disorder of MADs.

Taken together, meth-dependent behavior would cause the dissimilatory crossmodal on emotional processing, which would be a key step in the exploration of VA integration in psychiatry. In this paper, we explored the emotional VA integration disorder of MADs who only use meth among three experiments. Experiment 1 firstly adopted a synchronous audio–visual integration processing mode and examined whether VA integration with congruent emotion would show a higher ACC and shorter RT than single sensory cues (isolated face or voice) would, testing the enhancement effect of VA integration in HCs and MADs. Thus, the reduction in or absence of this effect would reflect impaired VA integration. We predicted that meth dependence might impair the VA integration in fear compared to HCs. Experiments 2 and 3 adopted asynchronous crossmodal mode including visual-leading and auditory-leading mode to test whether emotional cues would improve the emotional VA integration of MADs by the designs of sequential presentation of crossmodal cues. We assumed that the auditory cues would make a greater improvement than the visual cues in the crossmodal emotional integration of MADs. Additionally, to explore the influence of the duration of emotional cues on emotional VA integration, we presented shorter priming emotional cues in Experiment 2 and longer adapter priming ones in Experiment 3, respectively. With reference to existing studies, the priming emotional cues were presented for one time at around 500 ms (Shen et al., 2017), and the adapter cues were repeated four times, containing 100 ms of silence in around 3,176 ms (Kloth et al., 2010; Skuk and Schweinberger, 2013).

## Experiment 1

### Methods

#### Participants

Fifty male abstinent MADs (age: mean ± SD, 36.32 ± 8.43 years) were recruited from the Da Lian Shan Institute of Addiction Rehabilitation. MADs were subjected to a semi-structured clinical interview to exclude psychiatric or physical illness. Inclusion criteria were: (1) age range from 18 to 60 years old, (2) native Chinese and education experience was university education or lower, (3) no history of psychiatric or neurological disorder, (4) had been using purely meth for at least 1 year, with drug dosage of more than 2 g per month, and (5) abstinent for less than 3 months. Forty-five male HCs (32.98 ± 10.57 years) were carefully screened from Dalian. The demographic and clinical characteristics of MADs and HCs in Experiment 1 are given in Table 1.

**TABLE 1:** Demographic variables of the participants in Experiment 1 (mean ± SD).

Characteristics	HCs (*n* = 45)	MADs (*n* = 50)	*P*-value
Age (years)	32.98 ± 10.57	36.32 ± 8.43	0.090
Education (years)	10.40 ± 2.14	8.42 ± 2.52	< 0.001
Beck Depression Inventory	9.00 ± 7.39	15.48 ± 8.65	< 0.001
Beck Anxiety Inventory	26.69 ± 6.52	29.44 ± 9.64	0.111
Barratt Impulsiveness Scale	79.02 ± 17.39	85.28 ± 14.57	0.060
Pittsburgh Sleep Quality Index	5.31 ± 3.27	6.62 ± 3.81	0.077
Fagerström Test for Nicotine Dependence	3.64 ± 2.52	4.72 ± 2.22	0.030
Alcohol Use Disorder Identification Test	6.16 ± 5.82	6.64 ± 5.63	0.681
Dosage of meth use before abstinence (g/month)	NA	10.63 ± 12.46	NA
Years of meth use before abstinence	NA	8.42 ± 5.35	NA

*HC, healthy control; MAD, methamphetamine dependent; NA, not applicable.*

All participants were right-handed and had normal or corrected-to-normal vision. All of them provided written informed consent. The study was approved by the Ethics Committee of Liaoning Normal University and was conducted in accordance with the latest version of the Declaration of Helsinki.

#### Demographics and Clinical Measures

All participants were required to report their demographic information (age and education level) and complete the PSQI (Liu et al., 1996), AUDIT (Xue, 1989), BIS (Li et al., 2011), BDI (Beck et al., 1961), BAI (Beck et al., 1988), and FTND (Pan et al., 2010). MADs’ drug use history [years of meth use before abstinence and dosage of meth use before abstinence (g/month)] was also collected using a self-report survey (see Table 1).

#### Stimuli

Twenty facial pictures (ten neutral, ten fearful) were selected from the native Chinese Facial Affective Picture System (Gong et al., 2011), comprising half male and half female. The emotional valence of the facial pictures was significant [*t*(9) = 18.14, *p* < 0.001 (M ± SE, neutral 4.50 ± 0.04, fearful 2.57 ± 0.10)], and so was the arousal effect [*t*(9) = −19.02, *p* < 0.001 (neutral 3.13 ± 0.05, fearful 6.93 ± 0.23)]. The faces had the same size of 6.8 cm × 8.6 cm (6.5° × 8.2° of view angle). During the experiment, the participants needed to keep their eyes fixated on a central cross (0.75° × 0.75° of view angle).

The auditory stimuli (44.1 kHz sample rate, 16 bit) were emotional prosody (four neutral, four fearful) uttered by two males and two females taken from the Montreal Affective Voices database (Belin et al., 2008). The decibel of fearful and neutral sound was 83 ± 2.20 and 81.28 ± 2.92 dB (M ± SD), respectively. The frequency of fearful and neutral sound was 508.50 ± 286.79 and 160.00 ± 58.69 Hz, respectively. The original recordings were edited to approximately 719 ms (M ± SE, 719.11 ± 0.17 ms). The auditory stimuli were via two speakers placed in such a way that the sounds originated from the screen center. Each emotional VA has four prosody, and five face models were paired randomly within the same gender.

#### Procedure

**FIGURE 1:**
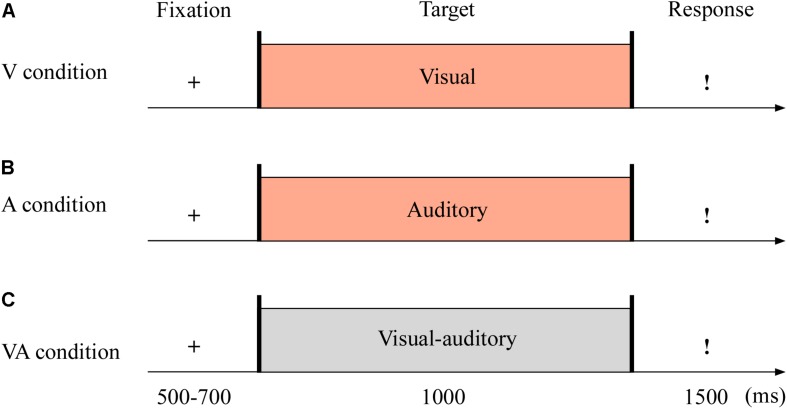
Sampled stimuli and procedure in Experiment 1. The procedure of Experiment 1 showed the sequence of events with a trial for visual sensory (V condition, **A**), auditory sensory (A condition, **B**), and visual–auditory crossmodal (VA condition, **C**) cues.

The targets were with either neutral or fearful emotion, yielding three modes: visual (V; see Figure 1A), auditory (A; see Figure 1B), and VA (see Figure 1C) stimuli. It was with 2 (emotion: neutral and fearful) × 3 (modality: V, A, and VA) design. All stimuli were set on a black background and presented on a 17″ LED monitor computer screen (with a refresh rate of 60 Hz and a spatial resolution of 1,280 × 768) at a viewing distance of 60 cm.

The experiment consisted of the practice phase (24 trials) and test phase (144 trials). The test phase included three blocks, and each block contained 48 trials. As shown in Figure 1, each trial began with a fixation cross varied from 500 to 700 ms. The target stimuli then appeared for 1,000 ms. Unless the stimulus was over, the participants could not report the emotion of the target as quickly as possible within 1,500 ms using the keyboard (1 = most fearful, 2 = less fearful, 3 = neutral).

#### Data Analyses

All statistical analyses were performed with SPSS 22.0 (IBM, Armonk, NY, United States). In Experiment 1, differences in the demographic and clinical measures between MADs and HCs were analyzed using independent-samples *t*-test. For the mean RT and ACC, we conducted a three-way repeated-measures ANOVA with 2 (emotion: neutral and fearful) × 3 (modality: V, A, and VA) × 2 (group: MADs and HCs). The *p*-values were corrected by Greenhouse–Geisser. We conducted Pearson correlation analyses to investigate the potential influence of BDI, FTND, and education years on behavioral indicators.

### Results

The demographics and clinical data of all participants in Experiment 1 are reported in Table 1. There were no group differences between HCs and MADs in age (*p* = 0.090), AUDIT score (*p* = 0.681), BAI score (*p* = 0.111), BIS score (*p* = 0.060), and PSQI score (*p* = 0.077). However, MADs scored higher in BDI (*p* < 0.001), FTND (*p* = 0.030), and the level of education (years) (*p* < 0.001) compared with HCs.

For the mean RT of the target, the main effect of modality [*F*(2,186) = 29.73, *p* < 0.001, ${\text{η}}_{\text{p}}^{2}$ = 0.242] was significant as it showed a much quicker RT in VA (mean ± SE: 507.53 ± 14.66 ms) and V (539.47 ± 16.15 ms) than A (580.02 ± 16.55 ms), all *p*-values < 0.001. The significant group effect [*F*(1,93) = 5.81, *p* = 0.018, ${\text{η}}_{\text{p}}^{2}$ = 0.059] revealed that HCs responded faster (506.56 ± 21.54 ms) than MADs (578.12 ± 20.43 ms). The main effect of emotion was not significant [*F*(1,93) = 0.08, *p* = 0.784, ${\text{η}}_{\text{p}}^{2}$ = 0.001].

**FIGURE 2:**
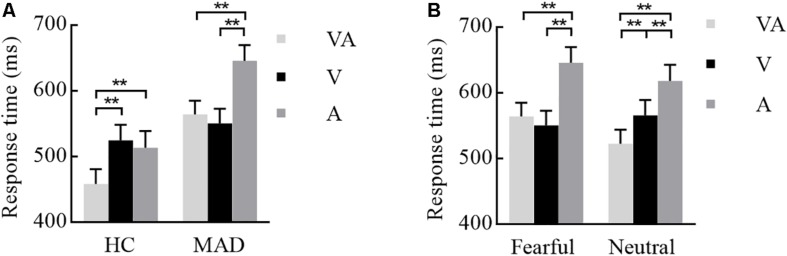
Combination of the results of Experiment 1. **(A)** Under a fearful emotion, a significant modality effect was noted in HCs and MADs. Results show a shorter RT for VA than A and V in HCs; however, there was no significant difference between VA and V in MADs. Besides, MADs had a longer RT than HCs in VA and A, not in V. **(B)** Under a neutral emotion, a significant modality effect was seen in MADs as a shorter RT for VA than A and V. ^∗∗^*p* < 0.01.

The interplay of groups by emotion and modality were significant [*F*(1,93) = 8.67, *p* = 0.004, ${\text{η}}_{\text{p}}^{2}$ = 0.087 and *F*(2,186) = 6.24, *p* = 0.005, ${\text{η}}_{\text{p}}^{2}$ = 0.063]. A three-way interplay of modality by emotion by group was significant [*F*(2,186) = 7.36, *p* = 0.002, ${\text{η}}_{\text{p}}^{2}$ = 0.073]. Further analysis suggested that, under fear, HCs required a longer RT for A (mean ± SE: 513.64 ± 25.18 ms) and V (524.79 ± 23.77 ms) than VA (458.89 ± 22.01 ms) (all *p*-values < 0.001) (see Figure 2A). MADs required a longer RT for A (646.02 ± 23.89 ms) than VA (564.43 ± 20.88 ms) and V (550.82 ± 22.55 ms) (all *p*-values < 0.001), but there was no significant difference between VA and V processing (*p* = 0.240) (see Figure 2A). On neutral, MADs required a longer RT for A (618.45 ± 24.29 ms) and V (565.99 ± 23.26 ms) than VA (522.99 ± 20.91 ms) (all *ps* < 0.001) (see Figure 2B). Under fear, the group showed a significant difference between VA and A (all *p*-values < 0.001) with a larger RT in HCs than MADs, not in V (see Figure 2A). However, under neutral VA, there was no significant difference between HCs than MADs (*p* = 0.200).

For the ACC of the target recognition, the significant emotion effect [*F*(1,93) = 14.82, *p* < 0.001, ${\text{η}}_{\text{p}}^{2}$ = 0.137] revealed that neutral (mean ± SE: 92.10% ± 1.60%) was higher than fearful (83.70% ± 0.21%). The modality effect [*F*(2,186) = 23.40, *p* < 0.001, ${\text{η}}_{\text{p}}^{2}$ = 0.201] reflected more effective outcome for VA (91.60% ± 1.60%) instead of V (88.40% ± 1.60%) and A (83.80% ± 1.70%). However, the group effect was not significant [*F*(1,93) = 1.28, *p* = 0.260, ${\text{η}}_{\text{p}}^{2}$ = 0.014]. There was no any other significant interplay (*p*-values > 0.05).

Pearson correlation analyses did not reveal a clear correlation pattern with the mean RT and ACC in six conditions for each group (see Supplementary Tables S1–S4), suggesting that, in general, these three variables (i.e., BDI, FTND, and education years) might not affect the present results.

### Discussion

The results of Experiment 1 showed that the crossmodal emotional disorder pertaining to fearful emotion was prevalent in MADs; that is, the RT for fearful emotion recognition in VA mode did not make a significant difference with V and A. However, this deficit was not found in neutral crossmodal processing, indicating that MADs did not have a wide range of VA integration disorder but only integrated obstacles for the fearful visual and auditory cues. In addition, during emotion processing of fear, we found that MADs had emotional recognition defects in either A or VA mode, but their visual ability was consistent with that of HCs. We suggested that the crossmodal emotional disorder in MADs might be related to their defects in auditory ability. According to the extension of the working model of emotional face–voice integration (Bruck et al., 2011), the process of emotional VA integration transferred the information from the primary sensory to the brain area responsible for integrated processing, and the information transmission could be directly between the visual and auditory primary cortex. Finally, the emotional identification of crossmodal integration was completed in the frontal region. Therefore, the impaired auditory perception ability may relate to the slow transmission of the auditory cortex, which caused reduced information transmission between the primary sensory cortex and the multisensory integrated brain region (Maurage et al., 2013). Moreover, the loss of information transmission among the integrated regions led to a weakened crossmodal integration. Ultimately, the dysfunction of the cognitive control loop (Payer et al., 2008; Kim et al., 2011) responsible for crossmodal emotion recognition led to a significant slowdown in the recognition of emotion.

In addition, during the crossmodal processing, there existed negative emotion deficits in MADs (Chen et al., 2018), indicating that MADs had the functional abnormality not only of the reflective system caused by cognitive control disorder but also of the emotional-automatic system responsible for emotional processing (Payer et al., 2008; Kim et al., 2011). From a neurological perspective, the crossmodal emotional disorder of MADs may be associated with the functional abnormalities of the frontal cortex and limbic areas (Canterberry et al., 2016).

Previous researches revealed that, with the crossmodal of facial and vocal, the visual and auditory stimuli at times appeared asynchronous. Under crossmodal integration, the anterior stimuli played the priming role (Stekelenburg and Vroomen, 2007), which prompted sensory systems to enter the readiness state and to speed up the response to later targets. Such prediction was likely related to the sensory modality of the cues (Cecere et al., 2017). Based on the prediction and promotion effect of the emotional cues during the crossmodal mode, Experiment 2 discussed whether the emotional recognition disorder of MADs could be improved through the designs of sequential presentation of crossmodal cues. We assumed that the emotional cues would improve the crossmodal emotional integration of MADs.

## Experiment 2

### Methods

#### Participants

Fifty-nine MADs (age: mean ± SD, 36.59 ± 8.43 years) were recruited from the Da Lian Shan Institute of Addiction Rehabilitation. Fifty-six HCs (34.00 ± 6.72 years) served as paid participants from Dalian. The selection criteria of the participants were as detailed in Experiment 1. The other details are also the same as in Experiment 1.

#### Demographics and Clinical Measures

The details were the same as in Experiment 1. The demographic and clinical characteristics of MADs and HCs in Experiment 2 are shown in Table 2.

**TABLE 2:** Demographic variables of the participants in Experiment 2 (mean ± SD).

Characteristics	HCs (*n* = 56)	MADs (*n* = 59)	*P*-value
Age (years)	34.00 ± 6.72	36.59 ± 8.43	0.072
Education (years)	10.60 ± 2.54	8.75 ± 3.44	0.001
Beck Depression Inventory	8.80 ± 7.02	16.73 ± 8.59	< 0.001
Beck Anxiety Inventory	25.29 ± 5.82	26.76 ± 6.33	0.197
Barratt Impulsiveness Scale	78.64 ± 19.23	85.19 ± 18.71	0.067
Pittsburgh Sleep Quality Index	4.96 ± 2.75	5.90 ± 3.35	0.106
Fagerström Test for Nicotine Dependence	3.64 ± 2.79	4.92 ± 2.46	0.011
Alcohol Use Disorder Identification Test	5.89 ± 6.34	6.64 ± 8.02	0.580
Dosage of meth use before abstinence (g/month)	NA	14.31 ± 27.40	NA
Years of meth use before abstinence	NA	7.15 ± 4.40	NA

*HC, healthy control; MAD, methamphetamine dependent; NA, not applicable.*

#### Stimuli

Twenty facial pictures (10 neutral, 10 fearful) were selected from the native Chinese Facial Affective Picture System (Gong et al., 2011), comprising half male and half female, the same as in Experiment 1. The auditory stimuli were emotional prosody (four neutral, four fearful) uttered by two males and two females taken from the Montreal Affective Voices database (Belin et al., 2008), the same as in Experiment 1.

#### Procedure

**FIGURE 3:**
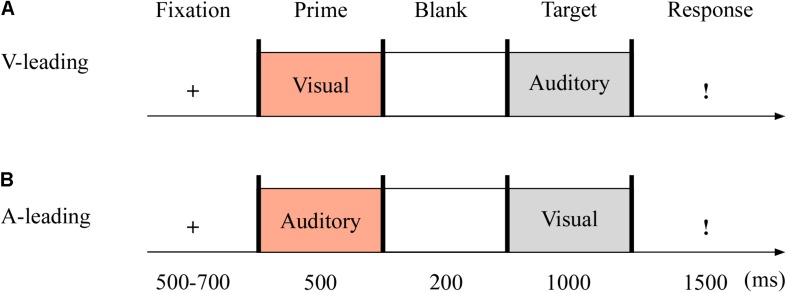
Sampled stimuli and procedure in Experiment 2. The procedure of Experiment 2 showed the sequence of events within a trial for visual-leading mode (V-leading, **A**) or auditory-leading mode (A-leading, **B**).

The targets were with either neutral or fearful emotion, yielding two modes: visual-leading (V-leading) and auditory-leading (A-leading). It was with 2 (emotion: neutral and fearful) × 2 (leading: V and A) design. The experiment consisted of the practice phase (16 trials) and test phase (96 trials). The test phase included eight blocks, with 12 trials in each block. The emotional valences of visual and auditory stimuli were consistent throughout. Each trial began with a fixation cross varied randomly for 500–700 ms. The emotional cues, either visual or auditory, were presented for 500 ms. After 200 ms of blank screen, the crossmodal stimuli then appeared for 1,000 ms. Unless the stimulus was over, participants could not report the emotion of the target as quickly as possible within 1,500 ms using the keyboard (1 = most fearful, 2 = less fearful, 3 = neutral) (see Figure 3).

#### Data Analyses

We mainly analyzed the mean RT and ACC using a three-way repeated-measures ANOVA with 2 (leading: V and A) × 2 (emotion: neutral and fearful) × 2 (group: MAD and HC) design. *P*-values were corrected by Greenhouse–Geisser. The other details are the same as in Experiment 1.

### Results

The demographics and clinical data of all participants in Experiment 2 are reported in Table 2. No significant group differences between HCs and MADs in age (*p* = 0.072), AUDIT score (*p* = 0.580), BAI score (*p* = 0.197), BIS score (*p* = 0.067), and PSQI score (*p* = 0.106) were found. However, compared with HCs, MADs scored higher in BDI (*p* < 0.001), FTND (*p* = 0.011), and the level of education (years) (*p* = 0.001).

For the mean RT of the target recognition, the main effect of leading was significant [*F*(1,113) = 21.29, *p* < 0.001, ${\text{η}}_{\text{p}}^{2}$ = 0.159] in that a quicker response was made in A-leading (mean ± SE: 474.92 ± 14.23 ms) than V-leading (510.18 ± 15.68 ms). The significant main effect of emotion [*F*(1,113) = 21.12, *p* < 0.001, ${\text{η}}_{\text{p}}^{2}$ = 0.157] revealed that the neutral (480.79 ± 14.33 ms) was processed in a rather shorter RT in comparison to fear (504.31 ± 15.06 ms). The significant group effect [*F*(1,113) = 9.60, *p* = 0.002, ${\text{η}}_{\text{p}}^{2}$ = 0.078] revealed that HCs responded faster (447.70 ± 20.74) overall than MADs (537.40 ± 20.20 ms).

**FIGURE 4:**
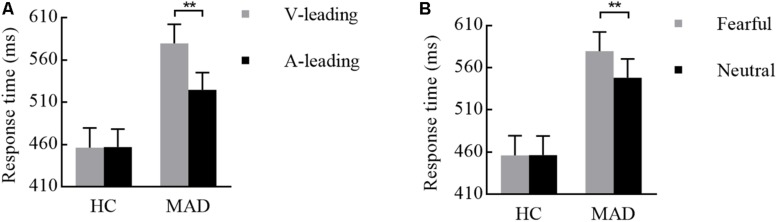
Combination of the results of RT for Experiment 2. **(A)** For fearful cues, MADs showed a longer RT in V-leading than that in A-leading cues, but HCs did not. **(B)** In V-leading cues, MADs showed a longer RT for neutral than that for fearful cues, but HCs did not. ^∗∗^*p* < 0.01.

Both two- and three-way interplays of leading by group and leading by emotion by group were significant, with *F*(1,113) = 5.38, *p* = 0.022, ${\text{η}}_{p}^{2}$ = 0.045 and *F*(1,113) = 4.82, *p* = 0.030, ${\text{η}}_{\text{p}}^{2}$ = 0.041, respectively. Under fearful, MADs displayed a rather shorter RT to A-leading (524.58 ± 20.65 ms) than V-leading (579.50 ± 22.88 ms) (*p* < 0.001; see Figure 4A), but there was no significant difference between A- and V-leading in HCs (*p* = 0.946). Under V-leading, RT to neutral (548.30 ± 22.12 ms) was typically shorter than fear (579.50 ± 22.88 ms) in MADs (*p* = 0.004). However, this phenomenon did not happen in HCs (*p* = 0.957; see Figure 4B). The two-way interplay of emotion by group was not significant [*F*(1,113) = 1.27, *p* = 0.262, ${\text{η}}_{p}^{2}$ = 0.011].

For the results of ACC, all main and interaction effects were not significant (*p*-values > 0.05).

Pearson correlation analyses did not reveal a clear correlation pattern with the mean RT and ACC in four conditions for each group (see Supplementary Tables S5–S8), suggesting that, in general, these three variables (i.e., BDI, FTND, and education years) might not affect the present results.

### Discussion

Compared to HCs, MADs particularly showed a slower response to the crossmodal emotional integration (Craparo et al., 2016). It might be related to the abnormal activation of prefrontal and other limbic-related regions involved in emotion and integration processing (Canterberry et al., 2016).

Interestingly, emotional recognition of crossmodal in MADs could be highly prompted by A-leading cues, especially the fearful sound, yet this dominance ceased to exist within HCs. It is suggested that the prediction of fearful sound played an important role in the crossmodal of MADs (Wang et al., 2017). This asymmetry between the visual- and auditory-leading cues suggested that there were separate, selectively recruited networks (Cecere et al., 2017). It has been suggested that the A-leading cues served as an alerting mechanism to boost visual processing immediately in crossmodal integration; in contrast, V-leading cues promoted the auditory system to make predictions about forthcoming auditory events (Thorne and Debener, 2014). Thus, our results suggested that auditory signs could activate the visual cortex faster to prepare for the incoming visual processing (Cecere et al., 2017) and improve the detection of facial emotions for MADs.

In addition, when given visually leading cues, MADs had a crossmodal disorder pertaining to fear. On the one hand, this may be due to the tendency of MADs to resist and avoid negative clues (Goldstein et al., 2002; Fox et al., 2005). On the other hand, the brain regions responsible for emotional cognitive processing in MADs were alienated or connected abnormally, and the decreased brain activity during processing of fear would lead to the slow processing of fear-inducing VA (Maurage et al., 2008). Overall, MADs were associated with crossmodal emotional disorder (Childress et al., 1999). The current results provided a new perspective for exploring the potential mechanisms of emotional disorder in MADs, and the predictive role of emotional cues may improve MADs’ ability to recognize emotions in the crossmodal mode.

The prediction effect is important for crossmodal (Jessen and Kotz, 2013). Generally, emotional adaptation played a prediction role in crossmodal (Wang et al., 2017). We shall now investigate whether emotional adaptation can highly promote crossmodal integration and whether the difference between V- and A-leading cues can provide more helpful predictions of crossmodal integration for MADs. All of these questions will be discussed in Experiment 3.

## Experiment 3

### Methods

#### Participants

Fifty-five male MADs (age: mean ± SD, 36.38 ± 8.52 years) were recruited from the Da Lian Shan Institute of Addiction Rehabilitation. Fifty-five HCs (33.71 ± 6.51 years) served as paid participants from Dalian. The other details are the same as in Experiment 1.

#### Demographics and Clinical Measures

The demographic and clinical characteristics of MADs and HCs in Experiment 3 are shown in Table 3.

**TABLE 3:** Demographic variables of the participants in Experiment 3 (mean ± SD).

Characteristics	HCs (*n* = 55)	MADs (*n* = 55)	*P*-value
Age (years)	33.71 ± 6.51	36.38 ± 8.52	0.067
Education (years)	10.51 ± 2.52	8.76 ± 3.52	0.003
Beck Depression Inventory	8.13 ± 6.72	16.65 ± 8.28	0.001
Beck Anxiety Inventory	25.09 ± 5.74	26.71 ± 6.58	0.172
Barratt Impulsiveness Scale	78.35 ± 19.86	84.80 ± 18.73	0.082
Pittsburgh Sleep Quality Index	5.02 ± 2.80	5.89 ± 3.31	0.139
Fagerström Test for Nicotine Dependence	3.61 ± 2.75	4.95 ± 2.39	0.008
Alcohol Use Disorder Identification Test	5.75 ± 6.07	6.93 ± 8.22	0.393
Dosage of meth use before abstinence (g/month)	NA	14.66 ± 28.33	NA
Years of meth use before abstinence	NA	7.11 ± 4.57	NA

*HC, healthy control; MAD, methamphetamine dependent; NA, not applicable.*

#### Stimuli

Twenty facial pictures (ten neutral, ten fearful) were selected from the native Chinese Facial Affective Picture System (Gong et al., 2011), comprising half male and half female, the same as in Experiment 1. The auditory stimuli were emotional prosody (four neutral, four fearful) uttered by two males and two females taken from the Montreal Affective Voices database (Belin et al., 2008), the same as in Experiment 1.

#### Procedure

**FIGURE 5:**
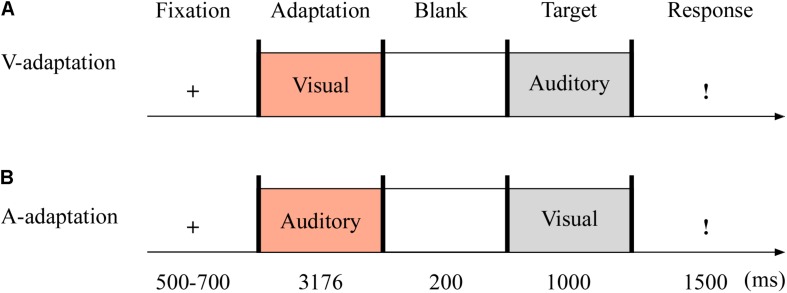
Sampled stimuli and procedure in Experiment 3. The procedure of Experiment 3 showed the sequence of events within a trial for visual-adaptation mode (V-adaptation, **A**) or auditory-adaptation mode (A-adaptation, **B**).

In Experiment 3 (see Figure 5), the auditory adaptation recordings were displayed for 3,176 ms, that is, the same four consecutive stimuli separated by three blank ones (i.e., 719 ms × 4 + 100 ms × 3). The visual cues in the adaptation stage were presented as long as the auditory stimuli. The other details are the same as in Experiment 2.

#### Data Analyses

The data analyses here are the same as in Experiment 2.

### Results

The demographics and clinical data of all participants in Experiment 3 are reported in Table 3. No group differences between HCs and MADs in age (*p* = 0.067), AUDIT score (*p* = 0.393), BAI score (*p* = 0.172), BIS score (*p* = 0.082), and PSQI score (*p* = 0.139) were observed. However, MADs scored higher in BDI (*p* < 0.001), FTND (*p* = 0.008), and the level of education (years) (*p* = 0.003) compared with HCs.

For the mean RT, the main effect of adaptation was significant [*F*(1,108) = 17.43, *p* < 0.001, ${\text{η}}_{\text{p}}^{2}$ = 0.139]. A quicker response was made in A-adaptation (mean ± SE: 496.28 ± 13.89 ms) than that in V-adaptation (534.38 ± 14.49 ms). The significant emotion effect [*F*(1,108) = 6.39, *p* = 0.013, ${\text{η}}_{\text{p}}^{2}$ = 0.056] revealed that a faster response was for neutral (508.39 ± 13.62 ms) than for fear stimuli (522.27 ± 13.82 ms). The significant group effect [*F*(1,108) = 16.91, *p* < 0.001, ${\text{η}}_{\text{p}}^{2}$ = 0.135] suggested that HCs (460.05 ± 19.01 ms) responded faster overall than MADs (570.60 ± 19.01 ms).

**FIGURE 6:**
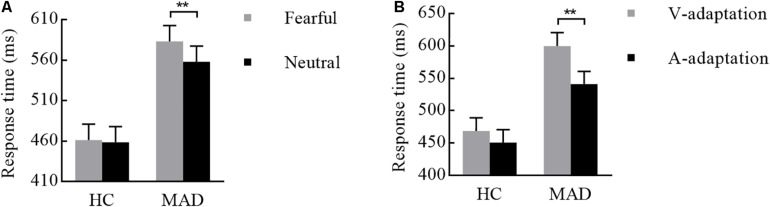
Combination of the results of RT for Experiment 3. **(A)** MADs showed a longer RT for neutral than for fearful cues, but HCs did not. **(B)** MADs showed a longer RT in V-adaptation than A-adaptation, but HCs did not. ^∗∗^*p* < 0.01.

There was a significant interplay of emotion by group [*F*(1,108) = 4.19, *p* = 0.043, ${\text{η}}_{\text{p}}^{2}$ = 0.037; see Figure 6A]. Further analysis suggested that emotion effect in MADs (*p* = 0.002) revealed a shorter RT to neutral (mean ± SE: 558.04 ± 19.26 ms) than fear (583.16 ± 19.54 ms). There was a significant interplay of adaptation by group [*F*(1,108) = 5.01, *p* = 0.027, ${\text{η}}_{\text{p}}^{2}$ = 0.044; see Figure 6B]. For MADs, RT to V-adaptation (599.86 ± 20.49 ms) was larger than to A-adaptation (541.34 ± 19.65 ms; *p* < 0.001). However, HCs did not show any significant difference between V-adaptation (468.89 ± 20.49 ms) and A- adaptation (451.22 ± 19.65 ms; *p* = 0.174).

For the analysis of ACC, the significant group effect [*F*(1,108) = 13.06, *p* < 0.001, ${\text{η}}_{\text{p}}^{2}$ = 0.108] suggested that MADs made a sharp increase (98.30 ± 1.20%) than HCs (92.20% ± 1.20%). However, the other main and interaction effects were not significant (*p*-values > 0.05).

Pearson correlation analyses did not reveal a clear correlation pattern with the mean RT and ACC in four conditions for each group (see Supplementary Tables S9–S12), suggesting that, in general, these three variables (i.e., BDI, FTND, and education years) might not affect the present results.

### Discussion

Experiment 3 found that, for MADs, crossmodal exhibited with faster RT in A-leading cues in comparison to V-leading cues. Either through A-adaptation (Experiment 3) or A-leading cues (Experiment 2), the prediction of emotional cues provided us a new perspective to better understand the relationship between drug addictive behavior and crossmodal integration. We suggest this finding revealed that if with visual cues MADs will have enough of an adaptation effect on the emotional context, it would promote crossmodal integration. That is, the V-leading emotion was sufficient to compensate for the given context, producing a strong prediction for auditory target and activating auditory areas to promote crossmodal integration. A basic relationship between prediction and crossmodal integration proposed in the present study was consistent with the results reported by Stekelenburg and Vroomen (2007). However, V-adaptation was unable to address the defects caused by slower visual perception in MADs (Vroomen and Keetels, 2010). This was consistent with previous results showing that A-leading cues could more easily induce crossmodal integration immediately, while V-priming was more difficult (van Atteveldt et al., 2007), especially for MADs.

In brief, this study provided novel findings that the prolonged presentation of visual cues might directly influence crossmodal integration. More importantly, for MADs, the crossmodal integration under A-leading cues had promoted emotional recognition ability, which to some extent compensated for the cognitive impairment of the facial emotion. Therefore, it could be speculated that the crossmodal emotional disorder of MADs may be related to the weakness of visual prediction.

## General Discussion

This study aimed to investigate the impact of meth dependents on the impairments of crossmodal processing. We observed the crossmodal deficits of fearful emotion in MADs. Additionally, individuals in MADs exhibited a promotion effect under the crossmodal emotional processing with auditory-leading cues compared to visual-leading cues. The crossmodal integration played adaptive roles in enriching perceptual representations and improving the reliability of emotional perception of MADs. However, knowledge about the mechanisms underlying the crossmodal deficiencies of MADs is still in its nascent stages. Now, the above three experiments were conducted to explore this mechanism.

In the present study, MADs showed a consistently poor performance in identifying fearful emotion. As we know, methamphetamine abuse could led to negative emotional states during withdrawal, like anxiety and depression behavior in mice (Ru et al., 2019). The results of BDI (see Tables 1–3) indicated that MADs were more likely to experience depression (Lai et al., 2015). This factor could account for their emotional deficiencies (Cox et al., 2018). MADs were more inclined to exhibit resistance and avoidance to negative cues (Goldstein et al., 2002; Fox et al., 2005), which could lead to an emotional disorder (Goldstein et al., 2002; Kim et al., 2011; Okita et al., 2016; Zhang L. et al., 2018). Li and Sinha (2008) suggested that drug dependence was largely mediated by dysfunction of the prefrontal and ACC regions. Thus, this dysregulation of cerebral mechanisms could motivate drug abuse (Goldstein and Volkow, 2011), related to the hypo-activity of the reflective system. The emotional dysregulation of MADs may be due to the hyper-activity of the affective-automatic system. Additionally, these brain abnormalities have been closely related to multisensory stimuli (Volkow et al., 2011b). The decreased activation of cognitive control circuits in the frontal region was likely to cause emotional disorder and cognitive or motivational dysfunction in people with addictions (Volkow et al., 2011a; Zhang L. et al., 2018) and caused the crossmodal emotional integration disorder as well (Yalachkov et al., 2012; Wang et al., 2018). The results of Experiment 1 were strongly consistent with our expectations that MADs showed poor fear recognition with crossmodal processing. Therefore, the present results preliminarily inferred that the potential causes of the emotional VA integration disorder of MADs may be due to the dysfunction of the top-to-bottom regulation of the higher cognitive system, as well as the imbalance between cognitive and emotional systems.

Combined with the results of Experiments 2 and 3, in MADs, crossmodal could be easily induced by A-leading cues (van Atteveldt et al., 2007), but slightly harder by V-leading ones. This verified that for MADs the visual cues could provide a strong predictive component for later auditory targets, which would then accelerate the crossmodal integration to compensate for the slower neural transduction of the primary visual cortex (Stekelenburg and Vroomen, 2007) and increase the difficulty of integration through V-leading cues (van Atteveldt et al., 2007). According to the neurobiological model of multisensory emotional information processing (Bruck et al., 2011), the emotional crossmodal integration would not be completed until the visual and auditory signs reached the frontal region. The results of Experiment 1 showed that MADs were with a weaker auditory ability, which caused the weak crossmodal integration. In Experiments 2 and 3, by the weaker priming effect of V-leading cues, the damaged auditory ability of MADs would make reduced information transmission. However, by the enhancement of the prediction effect of the auditory cues, the later visual signs made a more fluent transmission between the primary visual sensory cortex and the multisensory integrated brain region. Therefore, the current research confirmed that, in the asynchronous audio–visual integration mode, the crossmodal integration was related to not only the effectiveness of information transmission from the primary visual and auditory cortex but also the crossmodal prediction effect of the visual and auditory. For MADs, auditory cues could lead to a stronger predictability on the one hand and was also more conducive to the transmission of visual information to the integrated brain region.

Overall, the main purposes of this investigation were to explore the specificity of crossmodal disorder in MADs and present a description for the first time. The behavioral researches found associations between the damaged integrated ability and the brain dysfunction in individuals with addictions, which extended and updated the knowledge of crossmodal emotional integration in healthy people. Our findings provided evidence for the specific crossmodal integration of MADs which still awaits to be examined.

This was a preliminary exploratory study that provided an opportunity to understand the underlying neural mechanism of the crossmodal emotional disorder of MADs. More importantly, previous studies suggested that the crossmodal emotional integration disorder not only effectively predicted or evaluated the early stage of the development of substance addiction (Lannoy et al., 2017) but also improved the sensitivity of clinical diagnosis (Kajosch et al., 2016; Nan et al., 2018), which could be beneficial for explaining the relationship between crossmodal emotional dysfunction and clinical disease (Brandwein et al., 2015). This study offered a new suggestion for preventing and improving the emotional cognitive impairment of substance addictions as well as clinical interventions and treatment (Tinga et al., 2016; Nattala et al., 2018).

This study still has some limitations. Firstly, we only included male meth dependents. Given the robust gender differences in negative crossmodal processing (Gohier et al., 2013), women should be recruited in future research to enrich our findings. Secondly, we only made a simple observation indexed by RT and ACC but had not provided direct evidence to illustrate the relevant underlying neural mechanisms, which needed to be explored in the future.

## Conclusion

We found that crossmodal integration under long-term emotional adaptation filled the deficient emotion recognition for MADs, mainly through A-adaptation. We suggested that the emotional prediction effect of auditory or visual sensory could help with a better crossmodal and choosing a suitable strategy for these addictions in their developmental experiences of crossmodal integration and improving psychosocial outcomes. To sum up, the enhanced cognition for the complex crossmodal emotion could improve treatment outcomes and the quality of daily life and well-being of MADs.

## Data Availability Statement

All datasets generated for this study are included in the article/Supplementary Material.

## Ethics Statement

The studies involving human participants were reviewed and approved by Liaoning Normal University. The patients/participants provided their written informed consent to participate in this study.

## Author Contributions

ZZ conceived and designed the experiments, completed the specific data collection and analysis, and drafted and revised the research article critically. WH contributed significantly to modify and check the manuscripts and put forward some valuable and pertinent opinions. YL contributed to complete the specific data collection and analysis. MZ contributed to modify and check the manuscripts. WL proposed research directions and the completion of experimental design, article checking, and put forward specific ideas and methods to solve problems.

## Conflict of Interest

The authors declare that the research was conducted in the absence of any commercial or financial relationships that could be construed as a potential conflict of interest.

## Abbreviations

A: auditory
ACC: accuracy
ANOVA: analysis of variance
AUDIT: Alcohol Use Disorders Identification Test
BAI: Beck Anxiety Inventory
BDI: Beck Depression Inventory
BIS: Barratt Impulsiveness Scale
DLPFC: dorsolateral prefrontal cortex
FTND: Fagerström Test for Nicotine Dependence
HCs: healthy controls
MADs: methamphetamine dependents
meth: methamphetamine
OFC: orbitofrontal cortex
PSQI: Pittsburgh Sleep Quality Index
RT: response time
V: visual
VA: visual–auditory.
