# Supplementary material for: Facilitation of Crossmodal Integration During Emotional Prediction in Methamphetamine Dependents
Source: Front Neural Circuits. 2020 Jan 23;13:80. doi: 10.3389/fncir.2019.00080 (PMC6989411; doi:10.3389/fncir.2019.00080)
Supplement: Supplementary file 1 [file Data_Sheet_1.pdf]

Supplementary Material Supplementary Material

1 Supplementary Tables

Table S1. Pearson correlations between RT of Experiment 1 and some demographic characteristics in HCs, r (P-value).

	fearful			neutral		
	VA	V	A	VA	V	A
Education (years)	-0.141(0.355)	-0.140(0.358)	-0.183(0.229)	-0.163(0.285)	-0.150(0.327)	-0.009(0.952)
BDI	0.078(0.609)	0.131(0.392)	-0.030(0.844)	0.166(0.277)	0.119(0.438)	-0.079(0.607)
FTND	0.196(0.198)	0.265(0.079)	0.150(0.326)	0.139(0.362)	0.219(0.148)	0.127(0.404)

HCs: Healthy Controls; BDI, Beck Depression Inventory; FTND, Fagerström Test for Nicotine Dependence.

Table S2. Pearson correlations between RT of Experiment 1 and some demographic characteristics in MADs, r (P-value).

	fearful			neutral		
	VA	V	A	VA	V	A
Education (years)	-0.097(0.505)	-0.146(0.312)	-0.1(0.488)	-0.008(0.954)	-0.149(0.302)	-0.042(0.772)
BDI	0.123(0.395)	0.142(0.325)	0.125(0.386)	0.18(0.212)	0.229(0.109)	0.071(0.622)
FTND	-0.229(0.11)	-0.21(0.143)	-0.281(0.048)	-0.156(0.28)	-0.189(0.189)	-0.232(0.105)

MADs, Methamphetamine Dependents; BDI, Beck Depression Inventory; FTND, Fagerström Test for Nicotine Dependence.

Table S3. Pearson correlations between ACC of Experiment 1 and some demographic characteristics in HCs, r (P-value).

	fearful			neutral		
	VA	V	A	VA	V	A
Education (years)	-0.111(0.466)	-0.126(0.409)	-0.082(0.594)	-0.019(0.901)	-0.136(0.373)	-0.026(0.866)
BDI	-0.275(0.067)	-0.222(0.142)	-0.222(0.143)	-0.001(0.995)	-0.253(0.094)	-0.129(0.399)
FTND	0.157(0.254)	0.174(0.254)	0.129(0.399)	0.049(0.748)	0.066(0.669)	0.018(0.906)

HCs: Healthy Controls; BDI, Beck Depression Inventory; FTND, Fagerström Test for Nicotine Dependence.

Table S4. Pearson correlations between ACC of Experiment 1 and some demographic characteristics in MADs, r (P-value).

	fearful			neutral		
	VA	V	A	VA	V	A
Education (years)	0.142(0.324)	0.111(0.442)	0.187(0.193)	0.106(0.462)	0.041(0.776)	-0.094(0.517)
BDI	-0.129(0.373)	-0.164(0.255)	0.032(0.825)	-0.23(0.109)	-0.171(0.236)	-0.041(0.780)
FTND	0.124(0.392)	0.124(0.393)	0.202(0.160)	0.15(0.297)	0.158(0.274)	0.14(0.332)

MADs, Methamphetamine Dependents; BDI, Beck Depression Inventory; FTND, Fagerström Test for Nicotine Dependence.

Table S5. Pearson correlations between RT of Experiment 2 and some demographic characteristics in HCs, r (P-value).

	V-leading		A-leading	
	fearful	neutral	fearful	neutral
Education (years)	0.161(0.235)	0.148(0.278)	0.166(0.222)	-0.180(0.184)
BDI	0.098(0.471)	0.147(0.278)	0.079(0.562)	-0.114(0.401)
FTND	0.141(0.300)	0.105(0.440)	0.108(0.430)	-0.104(0.445)

HCs: Healthy Controls; BDI, Beck Depression Inventory; FTND, Fagerström Test for Nicotine Dependence.

Table S6. Pearson correlations between RT of Experiment 2 and some demographic characteristics in MADs, r (P-value).

	V-leading		A-leading	
	fearful	neutral	fearful	neutral
Education (years)	0.187(0.155)	0.118(0.373)	0.022(0.871)	0.026(0.846)
BDI	0.207(0.116)	0.225(0.086)	0.152(0.249)	0.095(0.474)
FTND	0.014(0.919)	0.078(0.559)	0.053(0.688)	0.006(0.966)

MADs, Methamphetamine Dependents; BDI, Beck Depression Inventory; FTND, Fagerström Test for Nicotine Dependence.

Table S7. Pearson correlations between ACC of Experiment 2 and some demographic characteristics in HCs, r (P-value).

	V-leading		A-leading	
	fearful	neutral	fearful	neutral
Education (years)	0.090(0.508)	0.037(0.789)	0.024(0.862)	-0.020(0.885)
BDI	0.035(0.800)	0.157(0.247)	0.006(0.966)	-0.139(0.307)
FTND	0.174(0.199)	0.217(0.108)	0.171(0.209)	-0.150(0.270)

HCs: Healthy Controls; BDI, Beck Depression Inventory; FTND, Fagerström Test for Nicotine Dependence.

Table S8. Pearson correlations between ACC of Experiment 2 and some demographic characteristics in MADs, r (P-value).

	V-leading		A-leading	
	fearful	neutral	fearful	neutral
Education (years)	0.233(0.075)	0.115(0.388)	0.180(0.173)	-0.169(0.201)
BDI	0.135(0.309)	0.012(0.927)	0.077(0.561)	-0.169(0.200)
FTND	0.120(0.364)	0.020(0.879)	0.106(0.424)	-0.004(0.978)

MADs, Methamphetamine Dependents; BDI, Beck Depression Inventory; FTND, Fagerström Test for Nicotine Dependence.

Table S9. Pearson correlations between RT of Experiment 3 and some demographic characteristics in HCs, r (P-value).

	V-adaptation		A-adaptation	
	fearful	neutral	fearful	neutral
Education (years)	0.207(0.129)	0.169(0.218)	0.043(0.757)	-0.103(0.456)
BDI	0.016(0.908)	0.124(0.366)	0.074(0.594)	-0.060(0.665)
FTND	0.210(0.124)	0.047(0.731)	0.117(0.394)	-0.133(0.334)

HC: Healthy Controls; BDI, Beck Depression Inventory; FTND, Fagerström Test for Nicotine Dependence.

Table S10. Pearson correlations between RT of Experiment 3 and some demographic characteristics in MADs, r (P-value).

	V-adaptation		A-adaptation	
	fearful	neutral	fearful	neutral
Education (years)	0.264(0.051)	0.153(0.264)	0.174(0.204)	0.211(0.123)
BDI	0.081(0.557)	0.128(0.353)	0.027(0.844)	0.029(0.834)
FTND	0.074(0.589)	0.043(0.758)	0.011(0.935)	0.057(0.679)

MADs, Methamphetamine dependents; BDI, Beck Depression Inventory; FTND, Fagerström Test for Nicotine Dependence.

Table S11. Pearson correlations between ACC of Experiment 3 and some demographic characteristics in HCs, r (P-value).

	V-adaptation		A-adaptation	
	fearful	neutral	fearful	neutral
Education (years)	0.058(0.674)	0.030(0.830)	0.067(0.625)	0.042(0.759)
BDI	0.110(0.424)	0.114(0.409)	0.066(0.633)	0.034(0.806)
FTND	0.191(0.163)	0.122(0.375)	0.183(0.180)	-0.153(0.265)

HC: Healthy Controls; BDI, Beck Depression Inventory; FTND, Fagerström Test for Nicotine Dependence.

Table S12. Pearson correlations between ACC of Experiment 3 and some demographic characteristics in MADs, r (P-value).

	V-adaptation		A-adaptation	
	fearful	neutral	fearful	neutral
Education (years)	0.108(0.434)	0.206(0.131)	0.055(0.689)	0.138(0.317)
BDI	0.100(0.467)	0.149(0.279)	0.072(0.603)	0.016(0.908)
FTND	-0.13(0.344)	0.141(0.304)	0.112(0.417)	-0.144(0.294)

MADs, Methamphetamine Dependents; BDI, Beck Depression Inventory; FTND, Fagerström Test for Nicotine Dependence.
